# Topical tofacitinib in treatment of *alopecia areata*

**DOI:** 10.31744/einstein_journal/2020AI5452

**Published:** 2020-08-06

**Authors:** Sineida Berbert Ferreira, Rachel Berbert Ferreira, Morton Aaron Scheinberg

**Affiliations:** 1 Clínica de Dermatologia Sineida Ferreira MaringáPR Brazil Clínica de Dermatologia Sineida Ferreira, Maringá, PR, Brazil.; 2 Centro Universitário de Maringá MaringáPR Brazil Centro Universitário de Maringá, Maringá, PR, Brazil.; 3 Hospital Israelita Albert Einstein São PauloSP Brazil Hospital Israelita Albert Einstein, São Paulo, SP, Brazil.

*Alopecia areata* is an autoimmune disease and the second most frequent cause of non-scarring hair loss. Several treatments have been described but are associated to low efficacy, particularly in the more resistant clinical forms. Janus kinase inhibitors are a new therapeutic option, and it may become the first line treatment in the next few years, as topical or oral use.^( 1 - 4 )^

**Figure 1:**
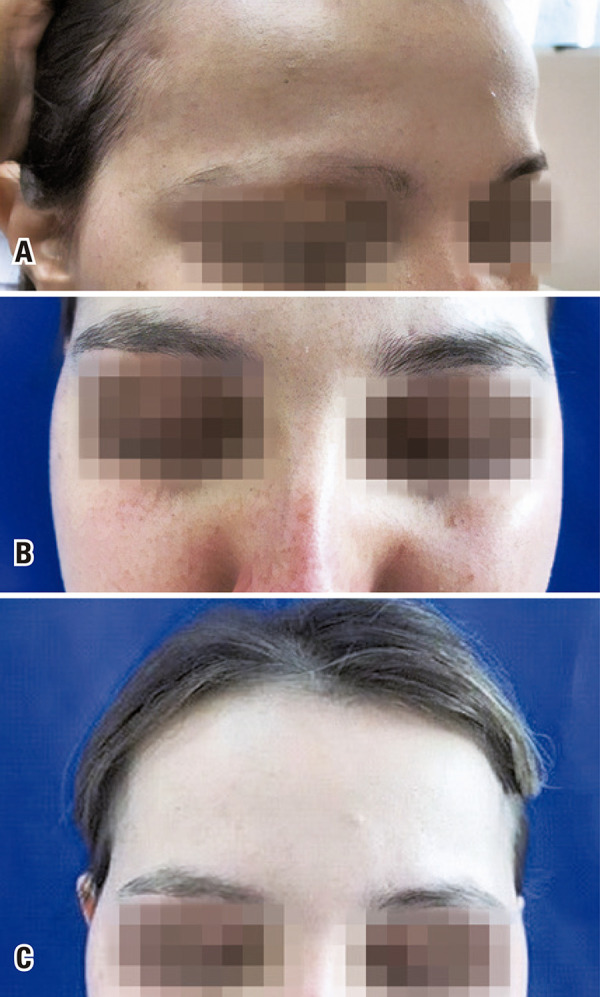
Recovery with topical therapy. (A) Before topical tofacitinib; (B) Two months after topical tofacitinib; (C) Four months after discontinuing topical tofacitinib

A 23-year-old female white female patient, with a history of *alopecia areata* for 12 years, presented diffuse hair loss in the scalp and part of the eyebrow. She had used oral steroids, cyclosporin and methotrexate, with no response on the right eyebrow ( Figure 1A ). She initiated on topical tofacitinib 2% (Chemistry Rx Compound and Specialty Pharmacy, Philadelphia, USA), once a day, and follow-up every 4 weeks. Hair growth was observed 4 weeks after initiating treatment ( Figure 1B ) and, two months later, there was complete hair growth of the eyebrow and of other alopecia sites. Four months after discontinuing the treatment, she maintains a positive response ( Figure 1C ).

*Alopecia areata* is a clinical manifestation of autoimmunity, presenting as hair loss, nails changes and association with other conditions, such as diabetes, vitiligo, rheumatoid arthritis and hypothyroidism (Hashimoto’s disease). It affects 2% of population. There are several subtypes of *alopecia areata* , and the most frequent forms are localized and *universalis* . It is known that infectious diseases, anxiety crises and hormone disorders may trigger its onset in genetically predisposed individuals, due to collapse of immune privilege of hair follicles. Experience with the topical use has been recently published by a team from Yale University, and our group has published many reports on oral use in the past 4 years.^( 5 - 9 )^ We believe the use herein reported is one of the first cases described in 2019.
