# Supplementary material for: Topical tofacitinib in treatment of alopecia areata
Source: Einstein (Sao Paulo). 2020 Aug 6;18:eAI5452. doi: 10.31744/einstein_journal/2020AI5452 (PMC7416648; doi:10.31744/einstein_journal/2020AI5452)
Supplement: Translation

# Translation: Tofacitinibe tópico no tratamento da alopecia areata

**Authors:** Sineida Berbert Ferreira, Rachel Berbert Ferreira, Morton Aaron Scheinberg

**Affiliations:** 1 Brazil Clínica de Dermatologia Sineida Ferreira, Maringá, PR, Brazil.; 2 Brazil Centro Universitário de Maringá, Maringá, PR, Brazil.; 3 Brazil Hospital Israelita Albert Einstein, São Paulo, SP, Brazil.

Alopecia areata é uma doença autoimune e a segunda causa de perda de cabelo não cicatricial. Várias propostas de tratamento foram descritas, mas estão associadas à baixa eficácia, em particular nas formas clínicas mais resistentes. Uma terapêutica com o uso dos inibidores do Janus quinase tem emergido como nova opção, sendo possível tornar-se o tratamento de primeira linha nos próximos anos, nas formas tópica ou oral.^( 1 - 4 )^

Uma paciente de 23 anos, de cor branca, com historia de 12 anos de alopecia areata apresentou perda difusa de cabelo e segmentos da sobrancelha. Fez uso prévio de corticosteroides orais, ciclosporina, metotrexato, porém sem resposta na sobrancelha direita ( Figura 1A ). Iniciou tofacitinibe tópico 2% (Chemistry Rx Compound and Specialty Pharmacy, Filadélfia, EUA), uma vez por dia e seguimento a cada 4 semanas. Foi possível observar repilação após 4 semanas do início do tratamento ( Figura 1B ) e, após 2 meses, completa repilação da sobrancelha e em outros locais com alopecia. Após 4 meses da suspensão do tratamento, manteve resposta positiva ( Figura 1C ).

**Figura 1:** Recuperação com terapia tópica. (A) Antes do tofacitinibe tópico; (B) 2 meses após tofacitinibe tópico; (C) 4 meses após suspender tofacitinib tópico

A alopecia areata é uma manifestação clínica de autoimunidade acometendo em torno de 2% da população com perda de cabelos, alterações ungueais e associação com outras condições clínicas como diabetes, vitiligo, artrite reumatoide e hipotireoidismo (Hashimoto). Existem vários subtipos de alopecia areata, sendo as formas localizada e universal as mais frequentes. Em indivíduos geneticamente predispostos, sabe-se que processos infecciosos, crises de ansiedade e distúrbios hormonais podem precipitar seu aparecimento, devido à perda de tolerância imunológica ao folículo piloso. Experiência com uso tópico foi recentemente publicado pelo grupo da *Yale University* , e várias publicações com o uso oral foram descritas pelo nosso grupo em publicações nos últimos 4 anos.^( 5 - 9 )^Acreditamos que o uso relatado seja um dos primeiros descritos em 2019.
